# Supplementary material for: gscreend: modelling asymmetric count ratios in CRISPR screens to decrease experiment size and improve phenotype detection
Source: Genome Biol. 2020 Mar 2;21:53. doi: 10.1186/s13059-020-1939-1 (PMC7052974; doi:10.1186/s13059-020-1939-1)
Supplement: Supplementary file 1 — Additional file 1 Table S1 and Figures S1–S4. [file 13059_2020_1939_MOESM1_ESM.pdf]

Additional file 1

gscreend: modelling asymmetric count ratios in CRISPR screens to decrease experiment size and improve phenotype detection

Katharina Imkeller, Giulia Ambrosi, Michael Boutros, Wolfgang Huber

Table S1: Comparison of different analysis tools for pooled CRISPR screens.

Tool	Statistical model for gRNA level	Statistical model for gene level
MAGeCK-RRA	negative-binomial distribution	robust ranking aggregation
BAGEL		Bayesian classification
ScreenBEAM	normal distribution	Bayesian hierarchical modeling
CRISPhieRmix	skew-normal distribution	hierarchical mixture model
CRISPRBetaBinomial (CB2)	beta-binomial distribution	Fisher's method
gscreend	skew-normal distribution	robust ranking aggregation

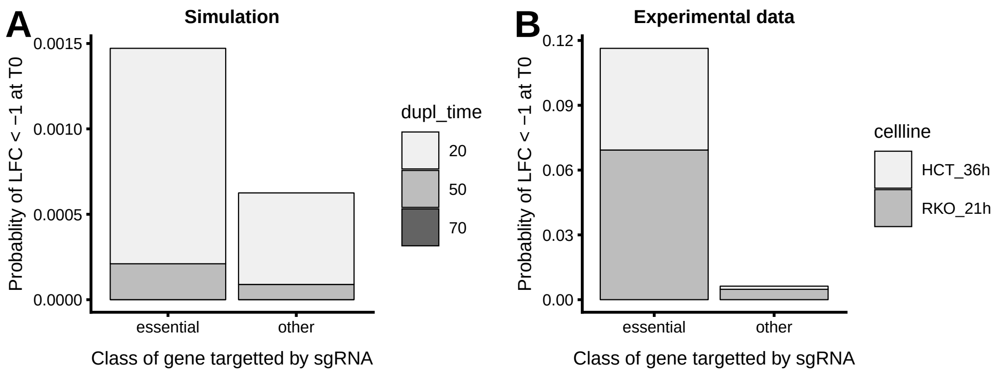

Fig. S1: Fraction of gRNAs with reduced abundance at T0. The class of gene targeted by each category of gRNAs is indicated. Essential genes in (A) according to Hart et al. (2017). In (B) essential genes are all those that belonged to the set of growth rate reducing genes. A: Simulation with doubling times of 20, 50 and 70 hours. B: Experimental data from RKO (doubling time 21h) and HCT116 cells (doubling time 36h).

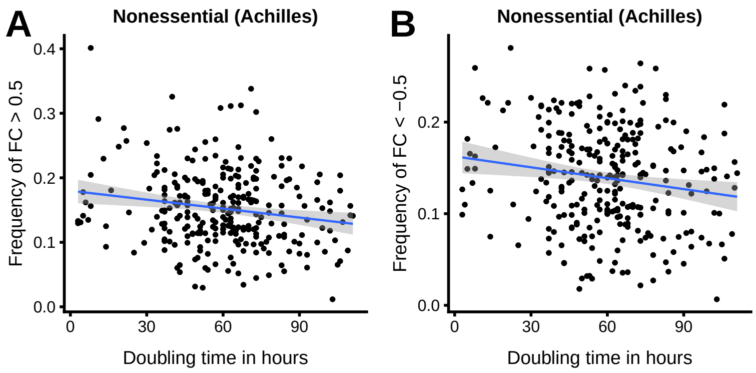

Fig. S2: Screening data from the DepMap project shows higher frequency of LFC at higher cell growth rate. Analyses were performed only for gRNAs targeting non-essential genes. A: Positive LFC>+0.5. B: Negative LFC<-0.5. (DepMap, 2019; Meyers et al., 2017; Tsherniak et al., 2017)

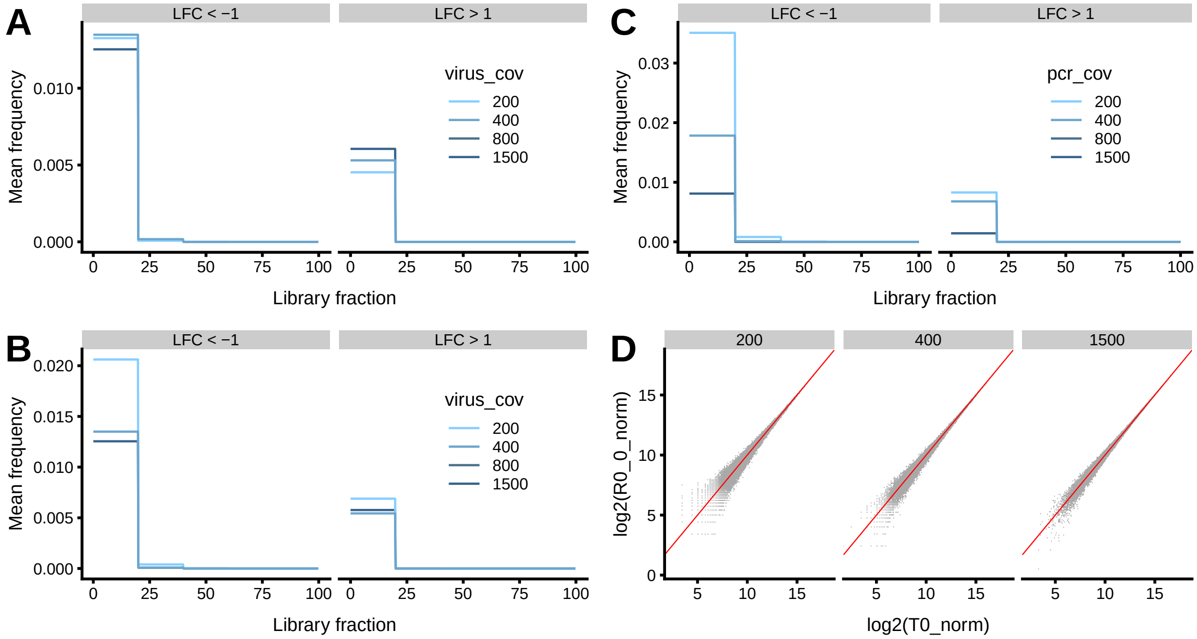

Fig. S3: Effect of changing transduction or PCR coverage is small compared to the effects described in Figure 2. A: Frequency of extreme LFC between library sequencing and T1 for different values of transduction coverage. B: Frequency of extreme LFC between T0 and T1 for different values of transduction coverage. C: Frequency of extreme LFC between T0 and T1 for different values of PCR coverage. D: log2 gRNA counts at T1 vs. T0 for different values of PCR coverage.

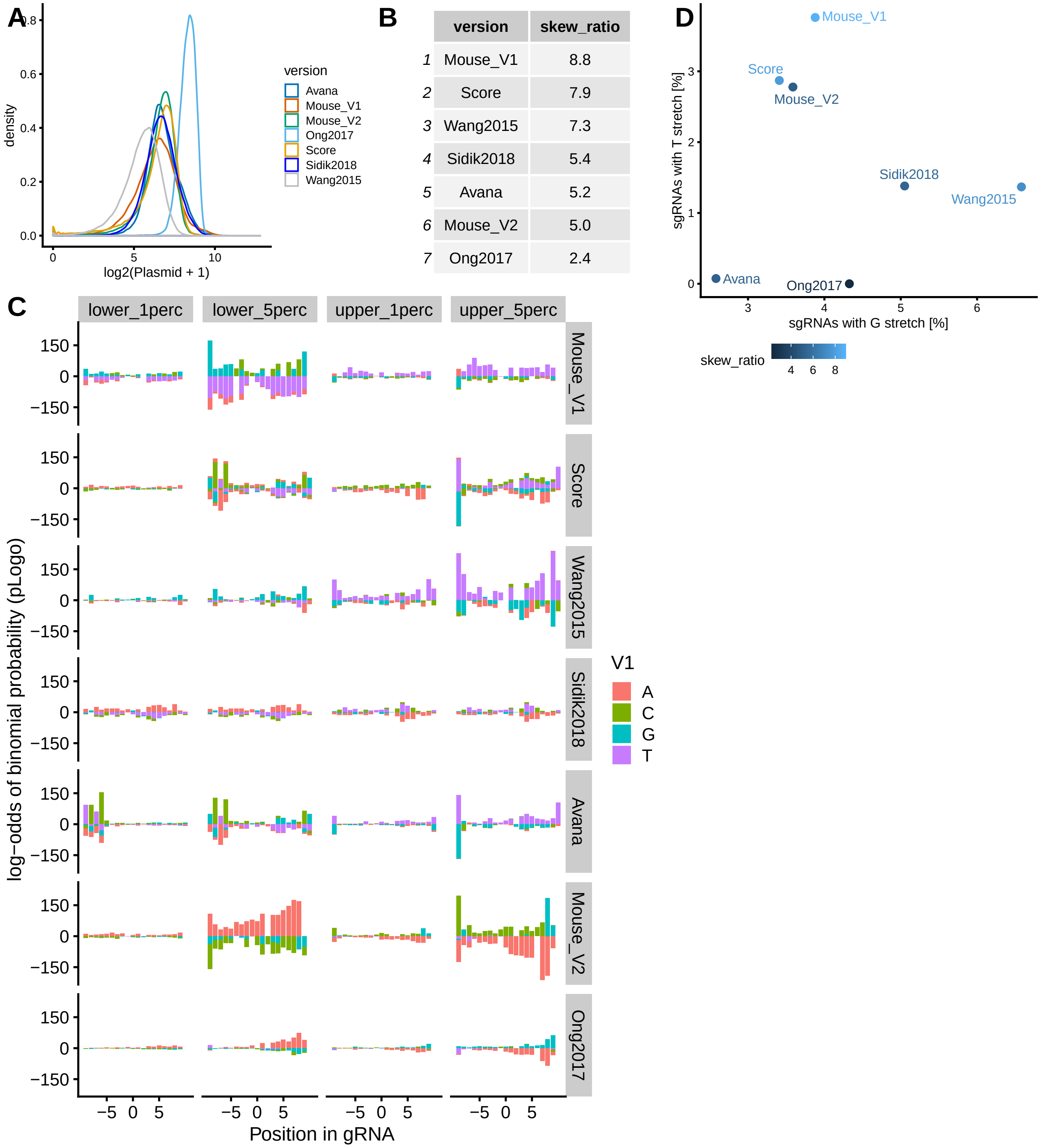

Fig. S4: gRNA sequence features influence homogeneity of library distribution. A+B: Selection of seven different CRISPR-KO libraries with different log2 gRNA count distributions (A, normalized to total counts) and library distribution widths (B) (DepMap, 2019; Meyers et al., 2017; Ong et al., 2017; Sidik et al., 2016; Tzelepis et al., 2016; Wang et al., 2015). C: Over- an underrepresented residues in gRNA sequence for 1 and 5 percent lower and higher abundant gRNAs in the libraries (ordered by decreasing distribution width). Log-odds of binomial probability were calculated using pLogo (O'Shea et al., 2013). D: Percentage of gRNAs with at least 4 consecutive Ts (y-axis) and at least three consecutive Gs (x-axis).

References

DepMap (2019). DepMap Achilles 19Q1 Public. Figshare *Fileset*.

Hart, T., Tong, A.H.Y., Chan, K., Van Leeuwen, J., Seetharaman, A., Aregger, M., Chandrashekhar, M., Hustedt, N., Seth, S., Noonan, A., et al. (2017). Evaluation and Design of Genome-Wide CRISPR/SpCas9 Knockout Screens. G3 Genes|Genomes|Genetics *7*, 2719–2727.

Meyers, R.M., Bryan, J.G., McFarland, J.M., Weir, B.A., Sizemore, A.E., Xu, H., Dharia, N.V., Montgomery, P.G., Cowley, G.S., Pantel, S., et al. (2017). Computational correction of copy number effect improves specificity of CRISPR–Cas9 essentiality screens in cancer cells. Nature Genetics *49*, 1779–1784.

Ong, S.H., Li, Y., Koike-Yusa, H., and Yusa, K. (2017). Optimised metrics for CRISPR-KO screens with second-generation gRNA libraries. Scientific Reports *7*, 1–10.

O'Shea, J.P., Chou, M.F., Quader, S.A., Ryan, J.K., Church, G.M., and Schwartz, D. (2013). PLogo: A probabilistic approach to visualizing sequence motifs. Nature Methods *10*, 1211–1212.

Sidik, S.M., Huet, D., Ganesan, S.M., Huynh, M.H., Wang, T., Nasamu, A.S., Thiru, P., Saeij, J.P., Carruthers, V.B., Niles, J.C., et al. (2016). A Genome-wide CRISPR Screen in Toxoplasma Identifies Essential Apicomplexan Genes. Cell *166*, 1423–1435.e12.

Tsherniak, A., Vazquez, F., Montgomery, P.G., Weir, B.A., Kryukov, G., Cowley, G.S., Gill, S., Harrington, W.F., Pantel, S., Krill-Burger, J.M., et al. (2017). Defining a Cancer Dependency Map. Cell *170*, 564–576.e16.

Tzelepis, K., Koike-Yusa, H., De Braekeleer, E., Li, Y., Metzakopian, E., Dovey, O.M., Mupo, A., Grinkevich, V., Li, M., Mazan, M., et al. (2016). A CRISPR Dropout Screen Identifies Genetic Vulnerabilities and Therapeutic Targets in Acute Myeloid Leukemia. Cell Reports *17*, 1193–1205.

Wang, T., Birsoy, K., Hughes, N.W., Krupczak, K.M., Post, Y., Wei, J.J., Lander, E.S., and Sabatini, D.M. (2015). Identification and characterization of essential genes in the human genome. Science *350*, 1096–1101.
