# Supplementary material for: gscreend: modelling asymmetric count ratios in CRISPR screens to decrease experiment size and improve phenotype detection
Source: Genome Biol. 2020 Mar 2;21:53. doi: 10.1186/s13059-020-1939-1 (PMC7052974; doi:10.1186/s13059-020-1939-1)
Supplement: Supplementary file 2 — Additional file 2 Review history. [file 13059_2020_1939_MOESM2_ESM.docx]

Review History

**First round of review**

**Reviewer 1**

**Were you able to assess all statistics in the manuscript, including the appropriateness of statistical tests used?**

Yes: I think the proposed methodology is reasonable.

**Were you able to directly test the methods?**

No

**Comments to author:**

In this manuscript Imkeller et al. present a novel statistical test for CRISPR screens and also provide its implementation in R package called gscreend.

I think the manuscript is well written and the authors address important problems for the design and analysis of CRISPR screens that were not fully explored such as asymmetry of the null distribution, optimal calculation of experimental size and why it is better to use the plasmid library as a reference.

These insights are important and of great interest for the genome editing community.

The only suggestion I have that could be dramatically improve this manuscript before publication is a comparison of gscreend with more recent tools that are currently not included:

1) https://genomebiology.biomedcentral.com/articles/10.1186/s13059-018-1538-6
2) https://genome.cshlp.org/content/29/6/999.full

Also, it may be helpful to briefly introduce the key ideas behind the competing methods presented.

**Reviewer 2**

**Were you able to assess all statistics in the manuscript, including the appropriateness of statistical tests used?**

Yes

**Were you able to directly test the methods?**

No

**Comments to author:**

Review: Modelling asymmetric count ratios in CRISPR screens to decrease experiment size and improve phenotype detection

This manuscript by Imkeller et al. describes how cells/guide, i.e. guide coverage as an experimental parameter in CRISPR screens influences screen results and data quality, and presents a new analytical approach for identifying genes with fitness effect at reduced experiment size. Reducing the number of cells in the initial population for screening, i.e. reducing experiment size by choosing a library with a narrower distribution is extremely useful for time and cost friendly experiment design. Therefore, I believe that study is a great contribution to the field, especially to the experimental side. There are few details that can easily be addressed.

First, this study is highly complementary to Nagy et al 2017 (CRISPUulator), which is cited only in passing. Nagy covers compelling arguments about major experimental design considerations such as library fold coverage and screen duration. Both the findings of Nagy and the improvements of Imkeller should be stated more explicitly throughout the manuscript.

Pg. 3. Re: “However, careful inspection of datasets shows that the distribution of the before/after ratios for negative controls is often asymmetric …” reference 34 (Daley et al. 2018).

Datasets involved in this study are from CRISPRi and CRISPRa screens. I understanding that you are using the conclusion of asymmetric ratio from this study as your baseline, and it is relevant, but I would suggest to make it clear that Daley et al. study is based on inference and activation datasets, while your study evolves around CRISPRko libraries and datasets.

In addition, this asymmetry is seems to be extremely weak; Fig 2G and S2 show very subtle differences. That doesn't invalidate the conclusions but it does suggest this is a problem that does not impart major bias into final results. It would behoove the authors to explicitly state this point: subtle effects, in combination with experimental factors such as library coverage, can reduce signal to noise.

Pg. 5. Re: Width of libraries influences data quality

Since authors already included 5 different CRISPR libraries in their investigation of how library width affects screen results and data quality, I would recommend including other available and widely used libraries (Avana and Score in particular) as well, to increase the significance of their finding. A colossal amount of Avana data has been available for some time now.

Furthermore, some commentary is appropriate about the interaction between library width and screen depth. E.g. narrow libraries need not be screened at the same coverage as wide ones to achieve the same S/N in results, because the left tail of low-abundance guides is smaller.

Pg. 6. Re: “… gscreend outperforms BAGEL, MAGeCK and screenBeam, especially within the 1000 lowest ranked genes (Fig. 4C).” also p6, "...five selected genes..."

It is standard practice in informatics papers to cherry pick examples that show why their method is superior and deserves to be published. In addition to this, could the authors demonstrate a set of screens performed under perhaps non-ideal experimental conditions where gscreend outperforms other methods? In other words, what is the experimental design regime where gscreend is the preferred method?

Minor points

Pg. 16. Re: gRNA sequence composition

It would be interest to see if and how these sequence logos change if looking at lower and upper 10% (or anywhere between 1-10%) of gRNAs, in addition to looking just at 1% of gRNAs.

Pg. 17. Re: “Gene level LFCs are calculated by averaging the LFCs over all gRNAs belonging to the gene.”

Curious if authors tried weighted averaging (weight – guide rank) and how it reflects on their results?

**Authors Response**

**Point-by-point responses to the reviewers’ comments:**

Reviewer #1

In this manuscript Imkeller et al. present a novel statistical test for CRISPR screens and also provide its implementation in R package called gscreend.

I think the manuscript is well written and the authors address important problems for the design and analysis of CRISPR screens that were not fully explored such as asymmetry of the null distribution, optimal calculation of experimental size and why it is better to use the plasmid library as a reference.

These insights are important and of great interest for the genome editing community.

The only suggestion I have that could be dramatically improve this manuscript before publication is a comparison of gscreend with more recent tools that are currently not included:

[https://genomebi ology.biomedcentral.com/articles/10.1186/s13059-018-1538-6](https://genomebiology.biomedcentral.com/articles/10.1186/s13059-018-1538-6)

<https://genome.cshlp.org/content/29/6/999.full>

*We thank the reviewer for their time and effort spent reviewing our work and for their constructive feedback. We also thank them for bringing these two recent tools to our attention. We included them in our analyses and comparisons summarized in the new Figure 4, and we updated the corresponding parts of the manuscript, in particular the Results and Methods section. Since the performance of these two newer tools was not superior to that of the previously included tools BAGEL and MAGeCK, we did not modify the more detailed follow-up regarding the ranking of specific genes in experimental data (4D), and the performance throughout varying experimental settings (4H, 4I).*

Also, it may be helpful to briefly introduce the key ideas behind the competing methods presented.

*We thank the reviewer for this suggestion. We have added Supplementary Table S1, which lists the six competing methods compared in our manuscript and the basic concepts behind each analysis tool. It is referenced accordingly in the Introduction as well as in the Results section related to Figure 4.*

Reviewer #2:

This manuscript by Imkeller et al. describes how cells/guide, i.e. guide coverage as an experimental parameter in CRISPR screens, influences screen results and data quality, and presents a new analytical approach for identifying genes with fitness effect at reduced experiment size. Reducing the number of cells in the initial population for screening, i.e. reducing experiment size by choosing a library with a narrower distribution is extremely useful for time and cost friendly experiment design. Therefore, I believe that study is a great contribution to the field, especially to the experimental side. There are few details that can easily be addressed.

First, this study is highly complementary to Nagy et al 2017 (CRISPUulator), which is cited only in passing. Nagy covers compelling arguments about major experimental design considerations such as library fold coverage and screen duration. Both the findings of Nagy and the improvements of Imkeller should be stated more explicitly throughout the manuscript.

*We thank the reviewer for their time and effort spent reviewing our work and for their constructive feedback. We agree that the study by Nagy et al. presents important considerations for experimental screen design, especially regarding the relationship of gRNA representation, screen duration and signal-to-noise ratio. We included two additional sentences to refer to these findings :*

*→ In the introduction, after having explained the concept of screening coverage and the varying experimental protocols, we state that Nagy et al. have implemented a computational simulation to model these choices.*

*→ In the discussion, we rephrased our previous reference to the study by Nagy et al. to better review (the relevant part of) their findings.*

Pg. 3. Re: “However, careful inspection of datasets shows that the distribution of the before/after ratios for negative controls is often asymmetric ...” reference 34 (Daley et al. 2018).

Datasets involved in this study are from CRISPRi and CRISPRa screens. I understand that you are using the conclusion of asymmetric ratio from this study as your baseline, and it is relevant, but I would suggest to make it clear that Daley et al. study is based on inference and activation datasets, while your study evolves around CRISPRko libraries and datasets.

*We reworded this paragraph to make it clear more immediately that the observation from Daley et al. was based on CRISPRi and CRISPRa screens, whereas our study looks at CRISPR-KO screens. However, we believe that the asymmetry is a general phenomenon that can be observed in CRISPR-KO, CRISPRi and CRISPRa screens in general. This does not exclude that there are other additional effects in CRISPRi and CRISPRa screens, like off-target effects, for example, that lead to an additional layer of complications.*

In addition, this asymmetry seems to be extremely weak; Fig 2G and S2 show very subtle differences. That doesn't invalidate the conclusions but it does suggest this is a problem that does not impart major bias into final results. It would behoove the authors to explicitly state this point: subtle effects, in combination with experimental factors such as library coverage, can reduce signal to noise.

*We agree that the difference shown in Fig. 2G and S2 look subtle, but these are intermediate views of the data. As a measure that is more relevant for the end user, and as one possible proxy of what the reviewer calls the final result, we suggest considering the recall. Indeed, Figure 2D shows substantial and non-negligible effects on phenotype detection: 10% of recall can get lost under realistic parameter assumptions.*

*The importance of taking into account the asymmetry is also supported by the results*

*shown in Figure 4H and 4I, which highlight that the recall strongly depends on the asymmetry (via splitting coverage and library skew ratio), all else being equal.*

Pg. 5. Re: Width of libraries influences data quality

Since authors already included 5 different CRISPR libraries in their investigation of how library width affects screen results and data quality, I would recommend including other available and widely used libraries (Avana and Score in particular) as well, to increase the significance of their finding. A colossal amount of Avana data has been available for some time now.

*We thank the reviewer for this excellent and helpful suggestion. Since it is not straightforward to directly compare phenotype detection across screens performed with different libraries and different cell lines, we used correlation between replicates as a measure of data quality. We added Figure 3F to show the relationship between library distribution width and replicate reproducibility in multiple different screens, including screens done with the Avana and Score libraries.*

*In addition, we included the Avana and the Score library in the revised version of Figure S4.*

Furthermore, some commentary is appropriate about the interaction between library width and screen depth. E.g. narrow libraries need not be screened at the same coverage as wide ones to achieve the same S/N in results, because the left tail of low-abundance guides is smaller.

*We thank the reviewer for this suggestion. The conclusion that narrow libraries need not be screened at the same coverage as wide ones is indeed one of the most important conclusions from our work. It is implied by Figure 4H, which also shows that the coverage necessary to maintain a certain signal-to-noise ratio can be reduced even further when using our analysis tool gscreend. To take up the reviewer’s suggestion, we added a sentence in Section “Implications for screen design”.*

Pg. 6. Re: “... gscreend outperforms BAGEL, MAGeCK and screenBeam, especially within the 1000 lowest ranked genes (Fig. 4C).” also p6, "...five selected genes..."

It is standard practice in informatics papers to cherry pick examples that show why their method is superior and deserves to be published. In addition to this, could the authors demonstrate a set of screens performed under perhaps non-ideal experimental conditions where gscreend outperforms other methods? In other words, what is the experimental design regime where gscreend is the preferred method?

*We would like to politely point out that the primary thrust of our methods comparison looks at global, non-cherry-picked measures including overall precision-recall (Fig. 4B) and ranking of a strong positive control, ribosomes (Fig. 4C), in real datasets. We had highlighted five selected genes to make the analysis more tangible and go beyond only global analyses, which to some readers can seem abstract. Looking at the 1000 lowest ranked genes is simply a pragmatic view on that part of the hit list that will be most important to the end user, since differences in ranking higher up the ranks are unlikely to be followed up.*

*The reviewer’s question for the experimental design regime where gscreend is the preferred method is addressed in Fig. 4H, where we use simulated data to showcase the scenarios where gscreend improves phenotype detection, as a function of different experimental parameters. To further respond to the reviewer’s suggestion, we added a new panel, Fig. 4I, which uses experimental data. It shows that gscreend is better or similar to MAGeCK, where the difference is greatest for screens performed with higher library distribution width and lower cell splitting coverage.*

*More generally, we believe that the expectation on authors' own comparisons in*

*conjunction with the presentation of a new method should be to show that the method can be better under some relevant conditions and measures of success. A fully unbiased evaluation, under all relevant conditions and measures of success according to community consensus, of multiple methods is usually a subsequent, and separate, process.*

Minor points:

Pg. 16. Re: gRNA sequence composition: It would be of interest to see if and how these sequence logos change if looking at lower and upper 10% (or anywhere between 1-10%) of gRNAs, in addition to looking just at 1% of gRNAs.

*We thank the reviewer for this suggestion, which we addressed in the new Figure S4. We also calculated the pLogo statistics for the lower and upper 5% of the libraries’ gRNAs. They are now displayed alongside the statistics for the lower and upper 1% of gRNAs in Figure S4C. As mentioned above, this analysis now also includes the Score and Avana libraries.*

Pg. 17. Re: “Gene level LFCs are calculated by averaging the LFCs over all gRNAs belonging to the gene.”: Curious if authors tried weighted averaging (weight – guide rank) and how it reflects on their results?

*Weighted averaging for the LFCs calculation is a very interesting idea. So far, we have not pursued it, since we are not aware of a generally agreed upon or provably optimal prescription for calculating the weights. Is there a publication that we could use to guide us? While it is plausible that a well-implemented approach to such weighting would improve results, we expect such benefits to be “additive” to the improvements by gscreend’s treatment of the asymmetry, i.e., it would generally improve results, but not change the ranking of the methods comparison considered here.*

**Second round of review**

**Reviewer 1**

I'm very satisfied with the revisions. I think the authors have addressed well the comments and the issues raised by myself and by the other reviewer. I am looking forward to see this published and I am happy to see the tool was already accepted in Bioconductor.

**Reviewer 2**

This revised manuscript artfully addresses my prior comments and, in my opinion, is strengthened. Moreover the acceptance of the software module into Bioconductor adds substantially to the relevance of this manuscript.
